# Comparative Mapping of N6-Methyladenine, C5-Methylcytosine, and C5-Hydroxymethylcytosine in a Single Species Reveals Constitutive, Somatic- and Germline-Specific, and Age-Related Genomic Context Distributions and Biological Functions

**DOI:** 10.3390/epigenomes9030035

**Published:** 2025-09-18

**Authors:** Thibaut Renard, Serge Aron

**Affiliations:** Evolutionary Biology and Ecology, Université Libre de Bruxelles, 1050 Brussels, Belgium; serge.aron@ulb.be

**Keywords:** DNA methylation, C5-methylcytosine, N6-methyadenine, C5-hydroxymethylcytosine

## Abstract

Background/Objectives: The DNA methylome allows environmental signals to be converted into stable and adaptive changes in gene expression. While 5-methylcytosine (5mC) has been extensively studied, alternative epigenetic marks such as N6-methyladenine (6mA) and 5-hydroxymethylcytosine (5hmC) remain poorly understood. Comparative studies of these marks are rare, and their results are often confounded by phylogeny, tissue type, developmental stage, or methodology. Here, we aimed to disentangle the constitutive, somatic- and germline-specific, and/or age-related patterns displayed by 6mA, 5mC, and 5hmC within a single species. Methods: We generated long-read nanopore sequencing data for somatic tissues of buff-tailed bumblebee (*Bombus terrestris*) males and their sperm, enabling simultaneous detection of 6mA, 5mC, and 5hmC. We used a stepwise approach to successively identify (i) constitutive patterns conserved between somatic tissues and sperm, (ii) differences between the soma and the germline, and (iii) age-related changes between young and old males. Results: We found distinct constitutive, somatic and sperm, and age-related specific signatures in the genomic contexts, maintenance fidelity, and biological functions associated with 6mA, 5mC, and 5hmC. Sperm cells consistently displayed lower methylation entropy than did somatic tissues, indicating more stable methylation patterns in the germline. 5mC exhibited the greatest variation across all genomic contexts; 6mA and 5hmC displayed less dramatic differences. The influence of age was subtler but revealed context-dependent remodeling of methylation, particularly for 5hmC. Conclusions: We observed that 6mA, 5mC, and 5hmC displayed constitutive, somatic- and sperm-specific, and age-related differences that were associated with distinct genomic contexts and biological functions, supporting the complementarity of these methylation marks and their diverging epigenetic roles.

## 1. Introduction

DNA methylation is an evolutionarily conserved epigenetic marking system used in the genomes of all taxa, from bacteria to mammals [1]. Different DNA methylation marks exist and co-occur. (i) N4-methylcytosine (4mC) is mostly found in the genomes of archaea and bacteria; it is involved in various aspects of DNA metabolism, including damage repair, genomic stability, and gene expression regulation [2,3]. (ii) C5-methylcytosine (5mC) is the most extensively studied and predominant epigenetic mark in eukaryotic genomes, where it plays a fundamental role in the regulation of gene expression [1]. (iii) C5-hydroxymethylcytosine (5hmC) is the first oxidation product during 5mC demethylation and was, thus, initially viewed as a mere byproduct. However, 5hmC is increasingly being recognized as a bona fide epigenetic mark that is involved in cell differentiation [4] and development [5] as well as in aging and disease progression [6]. (iv) N6-methyladenine (6mA) is the dominant epigenetic mark in bacterial genomes, where it is a key part of the restriction modification system [3,7,8]. 6mA was originally thought to be unique to prokaryotes [9], but its presence (albeit at low levels) has been confirmed in eukaryotic genomes, including those of fungi, plants, invertebrates, and mammals [10,11,12,13,14,15,16,17]. This mark is involved in diverse biological processes such as embryonic development [14,15,18], stress responses [19,20,21], and aging [22].

Despite the diversity in DNA methylation marks, most research has focused on 5mC. Other marks, such as 6mA and 5hmC, remain understudied, limiting our understanding of how they may compare to 5mC. Comparative studies have highlighted that 5mC, 6mA, and 5hmC are associated with distinct genomic contexts and biological functions [23]. For instance, 6mA and 5hmC appear to be important drivers of development and neurological processes [6,18], while 5mC is involved in a broader range of biological processes. However, epigenetic marks are highly dynamic and controlled by various interacting factors, meaning that the results of interspecies comparisons are often prone to various confounders, such as the underlying DNA sequence [24,25], tissue type [26], developmental stage [27,28], and age [29,30]. Methodological differences exacerbate these issues: while 5mC is typically detected via bisulfite sequencing, 6mA has historically been detected using mass spectrometry or restriction-based methods, which tend to over- and underestimate abundance, respectively [31,32].

Our study aims to address these limitations while comparing the genomic contexts and biological functions associated with 6mA, 5mC, and 5hmC. We used long-read nanopore sequencing (Oxford Nanopore Technologies [ONT]) to generate genome-wide, base-resolution maps of all three marks in the buff-tailed bumblebee (*Bombus terrestris*), a species in which 6mA, 5mC, and 5hmC occur in similar proportions across the genome. Because epigenetic patterns vary greatly with tissue type and age, we controlled for these factors by sequencing different tissues and cell types (somatic tissues vs. sperm cells) from individuals of different ages (young vs. old). We utilized a three-step analytical approach: (i) we identified constitutive patterns that were shared between somatic tissues and sperm across ages; we then sequentially discriminated (ii) between somatic tissues and sperm, and (iii) between ages in our comparative analyses. We found that 6mA, 5mC, and 5hmC occurred in different genomic contexts. Methylation entropy, a proxy for maintenance fidelity, differed among marks and genomic contexts. In addition, each mark was associated with different biological processes, molecular functions, and cellular components. Both tissue and age had a significant influence on mark genomic context and biological function. Collectively, these results suggest that the three marks each make a unique yet complementary contribution to genome regulation across somatic tissues and sperm cells of different ages, highlighting the importance of further exploring the roles of alternative epigenetic marks. Ultimately, such work should yield a comprehensive view of the entire DNA methylome.

## 2. Results

### 2.1. Constitutive Patterns

**Figure 1:**
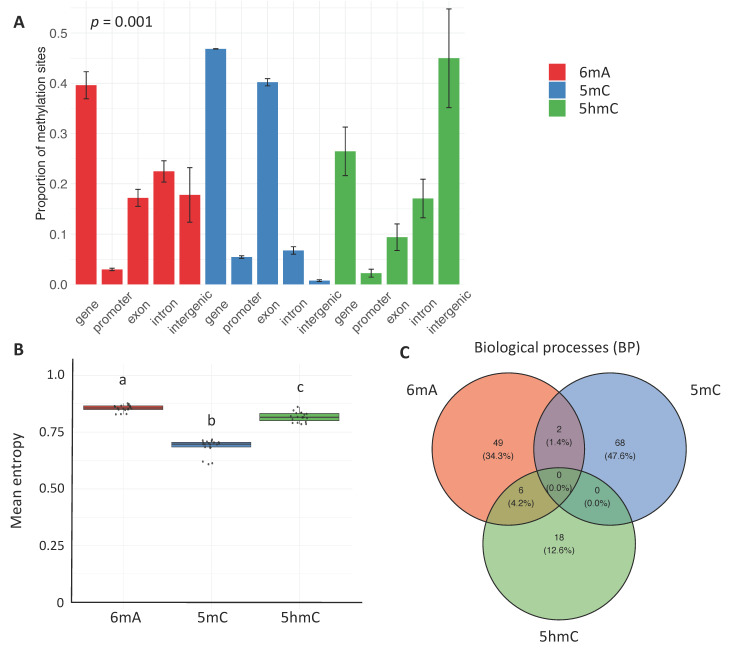
Constitutive patterns. (**A**) Genomic context. The barplot shows the proportions of the three epigenetic marks (mean ± SD) in different genomic contexts. Statistical differences in the composition of each methylation type across different genomic contexts were determined using permutational analysis of variance (PERMANOVA). Pairwise post hoc tests were used to evaluate whether the overall multivariate pattern of context proportions differs between two methylation types (adjusted *p*-value < 0.001 for all pairwise comparisons). (**B**) Methylation entropy. The line through the middle of each box is the median, and the lower and upper edges are the first and third quartiles, respectively. The different letters above the boxes indicate the presence of statistically significant differences (Kruskal–Wallis test: *p* < 0.05 and Dunn’s post hoc test: *p* < 0.05). (**C**) Biological functions. Venn diagram representing the number and percentage of GO terms associated with biological processes (BPs) that are unique to or shared by different marks. The GO terms for BP were considered to be significantly enriched (FDR < 0.05).

We started by broadly exploring constitutive patterns of 6mA, 5mC, and 5hmC by comparing the genomic distribution of each mark without considering tissue type or age. At the genome scale, each mark had a low percentage of significantly methylated sites (mean ± SD: 6mA = 2.9 ± 0.3%, 5mC = 1.9 ± 0.3%, 5hmC = 0.8 ± 0.2%), but these percentages differed significantly among marks (Kruskal–Wallis Chi-square = 48.9, df = 2, *p*-value = 2.5 × 10^−11^; Dunn post hoc test: adjusted *p*-value < 0.01 for each pairwise comparison). The distribution of the marks differed significantly among genomic contexts: 6mA largely occurred on gene bodies, 5mC on exons, and 5hmC on intergenic regions (PERMANOVA: df = 2, *p*-value = 0.001; post hoc tests: adjusted *p*-value < 0.001 for all pairwise comparisons; Figure 1A).

We then determined whether the three methylation marks displayed similar levels of maintenance fidelity by measuring methylation entropy, which expresses the variability of methylation at each site and serves as a proxy for epigenetic information content. More specifically, when methylation entropy is higher, there is greater variability in methylation levels [22,33,34], which results in lower stability. We found that, at the genome level, mean methylation entropy differed significantly among marks (mean ± SD: 6mA = 0.86 ± 0.02, 5mC = 0.69 ± 0.03, 5hmC = 0.82 ± 0.02; Kruskal–Wallis Chi-squared = 47.4, df = 2, *p*-value = 5.0 × 10^−11^; Dunn post hoc test: adjusted *p*-value < 0.01 for each pairwise comparison; Figure 1B). Additionally, for each mark, methylation entropy differed significantly among genomic contexts. Methylation entropy was highest for 6mA (exon = 0.83 ± 0.02, gene = 0.85 ± 0.01, intergenic region = 0.89 ± 0.01, intron = 0.86 ± 0.01, promoter = 0.84 ± 0.02; one-way ANOVA: df = 4, F = 55.5, *p*-value < 2 × 10^−16^, intermediate for 5hmC (exon = 0.81 ± 0.10, gene = 0.81 ± 0.03, intergenic region = 0.82 ± 0.03, intron = 0.81 ± 0.04, promoter = 0.84 ± 0.09; one-way ANOVA: df = 4, F = 122.9, *p*-value < 2 × 10^−16^), and lowest for 5mC (exon = 0.68 ± 0.03, gene = 0.69 ± 0.03, intergenic region = 0.74 ± 0.030, intron = 0.71 ± 0.04, promoter = 0.67 ± 0.03; Kruskal–Wallis Chi-square = 12.413, df = 4, *p*-value = 0.015) (Supplementary Figure S1). These findings indicate that methylation entropy was associated with both mark type and genomic context, highlighting context-specific differences in stability.

To examine whether 6mA, 5mC, and 5hmC are found on genes associated with different biological functions, we performed gene ontology (GO) enrichment analyses on biological processes (BPs), molecular functions (MFs), or cellular components (CCs). Using this approach, we could determine whether specific functional categories were overrepresented for genes carrying specific marks and, thus, identify the biological functions that are potentially being regulated. We found that the three marks differed in their GO enrichment patterns for BP, MF, and CC terms. Specifically, the BP analyses revealed that the top GO terms for 6mA were linked to transcription regulation and developmental processes, including neurogenesis and embryonic development. Those for 5mC were linked to proteostasis (protein transport, spliceosome complex, cytoplasmic translation) and DNA repair, and those for 5hmC were linked to transcription regulation and neurodevelopment. The MF analyses revealed that the top GO terms for 6mA and 5mC were associated with diverse binding activity (protein, chromatin, ATP, transcription factor, kinase, serine/kinase), while those for 5hmC were associated with transcriptional regulation as well as cell adhesion and signaling. The CC analyses revealed that the top GO terms for 6mA were linked to protein products mostly found in the cytosol and the nucleoplasm, those for 5mC were linked to protein-producing and trafficking organelles (nucleus, ribosome, Golgi apparatus), and those for 5hmC were linked to neural structures and membrane-associated domains. The marks had unique sets of significantly enriched GO terms (Figure 1C). However, some terms were enriched for both 6mA and 5mC, and for 6mA and 5hmC, but not for 5mC and 5hmC. 6mA and 5hmC more commonly shared BP terms, among which some were related to transcriptional regulation and neurogenesis, while 6mA and 5mC more commonly shared MF (chromatin binding, kinase activity, and transcriptional regulation) and CC (nucleus) terms. This result suggests that 6mA and 5mC may both help regulate processes occurring within the nucleus, despite their largely distinct genomic distributions.

Taken together, these results show that the three marks differed in their constitutive patterns: they were found in distinct genomic contexts and had unique sets of significantly enriched GO terms associated with biological functions.

### 2.2. Somatic- vs. Germline-Specific Signatures

**Figure 2:**
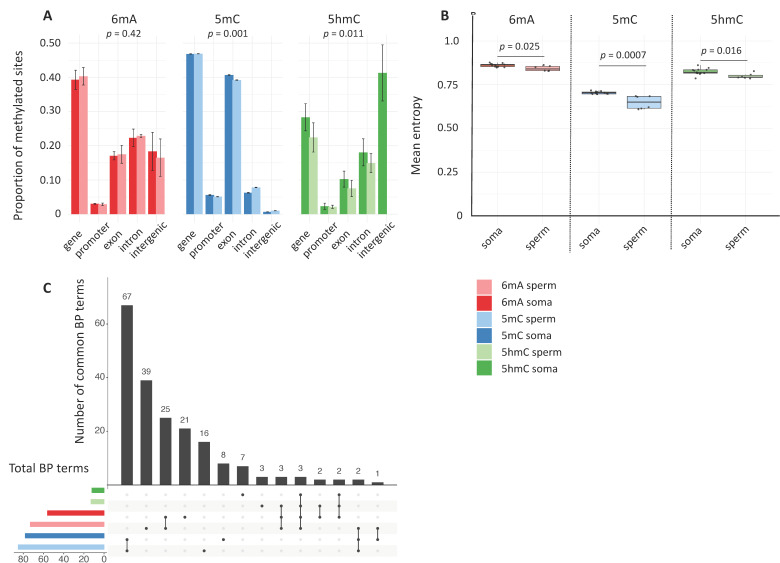
Somatic and germline differences. (**A**) Genomic context. The barplot shows the proportions of the three epigenetic marks (mean ± SD) in the soma vs. sperm in different genomic contexts. Statistical differences between the two sample types across the genomic contexts were determined for each mark using permutational analysis of variance (PERMANOVA). (**B**) Methylation entropy. The line through the middle of each box is the median, and the lower and upper edges are the first and third quartiles, respectively. Statistical significance was calculated with Wilcoxon Mann–Whitney tests. (**C**) Common GO terms associated with biological functions. The X axis indicates the total number of significantly enriched GO (BP) terms (FDR < 0.05) for the different combinations of mark and sample type (somatic tissues vs. sperm cells); the Y axis shows the number of these terms that were shared between combination categories (black dots).

Next, we explored differences in 6mA, 5mC, and 5hmC between the soma and the germline by incorporating two types of samples (somatic tissues vs. sperm cells) in the previously described analytical framework. Genome-wide methylation levels did not differ between somatic tissues and sperm cells for any of the marks (Kruskal–Wallis Chi-square = 2.2, df = 1, *p*-value = 0.14). However, there was a significant effect of tissue type on genomic context for 5mC (PERMANOVA: *p*-value < 0.001) and, to a lesser extent, for 5hmC (PERMANOVA: *p*-value = 0.01); there was no effect for 6mA (PERMANOVA: *p*-value = 0.42) (Figure 2A).

We compared methylation entropy between somatic tissues and sperm cells for each mark to determine if maintenance fidelity differs between the soma and the germline. Mean methylation entropy was significantly lower in sperm cells than in somatic tissues for all three marks (Wilcoxon Mann–Whitney: *p*-value < 0.05 for all pairwise comparisons; Figure 1B). This difference was most pronounced for 5mC (−7.8%) and was much smaller for 6mA and 5hmC (−2.2% and −3.1%, respectively). Differences in methylation entropy varied across genomic contexts. Notably, when a difference was detected, methylation entropy was always lower in sperm than in the soma, indicating higher stability in the germline. Specifically, for 6mA, methylation entropy was significantly lower in sperm cells than in somatic cells for exons, gene bodies, and promoters (Wilcoxon Mann–Whitney: *p*-values < 0.01), while no difference was seen for introns and intergenic regions. For 5mC, methylation entropy was consistently lower in sperm cells than in somatic cells across all the genomic contexts: exons, introns, promoters, and gene bodies (all *p* < 0.001) as well as intergenic regions (*p*-value = 0.002). For 5hmC, the pattern was more variable: methylation entropy was lower in sperm cells than in somatic cells for gene bodies (*p*-value = 0.022) and intergenic regions (*p*-value = 0.037), but there was no difference for exons, introns, or promoters (Supplementary Figure S2).

GO enrichment analyses revealed that, for all three marks, somatic cells and sperm cells shared a large percentage of significantly enriched terms related to BP, MF, and CC (Figure 2C). The remaining GO terms were generally functionally related to one another (see Results—Section 2.1).

Overall, we found that, while methylation patterns and maintenance fidelity differed between sperm cells and somatic cells, 6mA, 5mC and 5hmC functional roles appeared to be largely conserved between the soma and the germline.

### 2.3. Age-Related Changes

**Figure 3:**
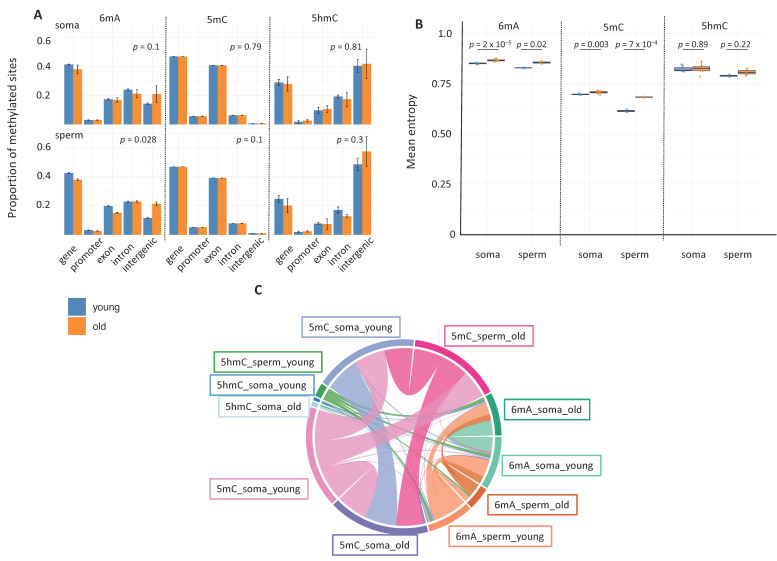
Age-related differences. (**A**) Genomic context. The barplot shows the proportions of the three epigenetic marks (mean ± SD) in different genomic contexts. Statistical differences between the two age groups across the genomic contexts for each mark and sample type were determined using permutational analysis of variance (PERMANOVA). The influence of cell type and age were tested separately. The influence of sample type was significant for 5hmC (R^2^ = 0.32, *p* = 0.010) and 5mC (R^2^ = 0.96, *p* = 0.001) but not for 6mA (R^2^ = 0.02, *p* = 0.43). The influence of age was significant for 6mA (R^2^ = 0.45, *p* = 0.002) but not for 5hmC (R^2^ = 0.05, *p* = 0.30) or 5mC (R^2^ = 0.005, *p* = 0.62). (**B**) Methylation entropy. The line through the middle of each box is the median, and the lower and upper edges are the first and third quartiles, respectively. Statistical significance was determined using a Student’s *t* test, Welsh test, or Wilcoxon Mann–Whitney test depending upon whether assumptions of normality and heteroscedasticity were met. (**C**) Age-related GO enrichment patterns. Chord diagram showing the significantly enriched terms (FDR < 0.05) related to BP that were shared by the two age groups. Each arc represents a specific combination of mark, sample type, and age. The chords convey the strength of the connections between the arcs, where chord width is positively related to the relative number of shared terms, and chord shade is positively related to the connection’s statistical significance.

We examined age-related changes in 6mA, 5mC, and 5hmC patterns by further refining our analyses: we discriminated between age groups (young: 1-week-old males vs. old: 5-week-old males) within the soma and the germline. We chose age groups representing the two extremes of the adult lifespan of *B. terrestris* males with a view to obtaining the strongest age-related signal possible under laboratory conditions [22]. Age did not influence genome-wide methylation levels in the soma and the germline for any of the marks (6mA: Kruskal–Wallis Chi-square = 2.53, df = 3, *p*-value = 0.47; 5mC: Kruskal–Wallis Chi-square = 2.13, df = 3, *p*-value = 0.55; 5hmC: Kruskal–Wallis Chi-square = 1.50, df = 3, *p*-value = 0.68). In contrast, age did significantly influence genomic context for 6mA (PERMANOVA: *p*-value = 0.02) but not for 5mC (PERMANOVA: *p*-value = 0.62) or 5hmC (PERMANOVA: *p*-value = 0.30) independently of sample type (Figure 3A). When somatic tissues and sperm cells were considered separately, the effect of age on 6mA was significant in the soma (*p* = 0.03) but not in sperm (*p* = 0.1), whereas no significant age changes were observed for 5mC (soma: *p* = 0.79, sperm: *p* = 0.1) or 5hmC (soma: *p* = 0.81, sperm: *p* = 0.3).

We also analyzed somatic and sperm age-related differences in methylation entropy. Age-related differences in methylation entropy varied based on mark and sample type. For 6mA and 5mC, methylation entropy differed between young and old individuals for somatic cells (Student’s *t*-test: *p*-value = 2 × 10^−5^ for 6mA and *p*-value = 0.003 for 5mC) and for sperm cells (Student’s *t*-test: *p*-value = 0.02 for 6mA and *p*-value = 7 × 10^−4^ for 5mC). No such age-related difference was observed for 5hmC (Student’s *t*-test: *p*-value = 0.89 for somatic cells, *p*-value = 0.22 for sperm cells), indicating that 5hmC methylation patterns may be more stable over the course of aging (Figure 3B). We also examined how these differences showed up across different genomic contexts. More specifically, for 6mA in somatic cells, methylation entropy displayed age-related differences for gene bodies, intergenic regions, and introns (Wilcoxon Mann–Whitney or *t*-tests: *p*-values < 0.005 for all pairwise comparisons) but not for exons or promoters. In contrast, for 6mA in sperm cells, methylation entropy displayed age-related differences for exons, gene bodies, intergenic regions, and introns (*t*-tests and Welch *t*-tests: *p*-values < 0.05) but not for promoters. This latter result highlights that age influenced 6mA methylation in most genomic contexts for sperm cells. For 5mC, age influenced methylation entropy across all five genomic contexts in both somatic cells (all *p*-values < 0.05) and sperm cells (all *p*-values < 0.001). In contrast, for 5hmC, there were no significant age-related differences in methylation entropy with the exception of those seen for the intergenic regions of sperm cells (*t*-test: *p* = 0.038) (Supplementary Figure S3).

The GO enrichment analyses showed that age had a significant influence on the enrichment of BP terms (Figure 3C). For 6mA in somatic cells, there were more significantly enriched GO terms for the young group than for the old group, but a moderate number of terms were shared. For 6mA in sperm cells, a similar age-related difference was observed, but a smaller number of terms were shared by the two age groups, indicating more age-related differences. For 5mC in both somatic cells and sperm cells, young and old individuals had similar numbers of significantly enriched GO terms and displayed a large degree of overlap, suggesting greater stability in methylation patterns over the course of aging. For 5hmC in somatic cells, very few GO terms were significantly enriched in either age group, and there was minimal overlap. For 5hmC in sperm cells, significantly enriched GO terms were seen in the young group but not in the old group, suggesting an age-related loss in 5hmC-associated functions.

Overall, we observed pronounced age-related differences in methylation entropy in somatic cells and sperm cells for 6mA and 5mC but not for 5hmC. It is important to note that the somatic- and sperm-specific GO terms with age-related enrichment patterns were generally related to similar functions. This finding suggests that each mark is involved in complementary regulatory mechanisms that likely help maintain biological consistency despite the above differences.

## 3. Discussion

This study compared methylation patterns for three epigenetic marks—6mA, 5mC, and 5hmC—within a single species, the buff-tailed bumblebee (*Bombus terrestris*). We uncovered distinct constitutive, somatic- and germline-specific, and age-related patterns in genomic context, maintenance fidelity, and biological functions for each mark. Our findings provide robust evidence about the complementarity of each mark within the DNA methylome. Thus, conducting analyses of multiple DNA methylation types may offer deeper insights into the interaction between the DNA methylome and systemic biological processes, such as development, the stress response, and aging.

We observed that the three marks were associated with different genomic contexts, indicating divergence in their targets and, potentially, in their functional roles. 6mA was predominantly found on gene bodies (introns and exons), which is consistent with what has been seen across taxa of animals [35], plants [36], and fungi [37]. 5mC was also mainly found on gene bodies but namely on exons, the typical pattern seen in mosaic DNA methylomes as reported for *B. terrestris* [38]. However, 5hmC was primarily found on intergenic regions, which contrasts with what has been seen in mammals, where 5hmC mostly occurs on gene bodies [39,40,41]. That said, it is worth noting that, in mammals, local 5hmC peaks occur in regulatory intergenic regions (e.g., enhancers) and help regulate gene expression [39,40,41]. The genomic distribution of 5mC and, to a lesser extent, that of 5hmC, but not 6mA, differed significantly between the soma and the germline. This finding may suggest that 5mC and 5hmC are involved in maintaining epigenetic patterns specific to the soma or the germline, whereas 6mA may play stable, conserved roles across both. A caveat is that we explored these patterns in adult individuals, not in developing individuals, and 6mA is known to have important regulatory functions in insects and mammals during development [18,42]. Thus, expanding our analyses to developmental stages may reveal more pronounced variations in 6mA. On that note, there were marked age-related differences in the genomic contexts of 6mA but not those of 5mC and 5hmC. The distinct constitutive, soma- and germline-specific, and age-related differences in the genomic distributions of 6mA, 5mC, and 5hmC highlight the existence of a complex DNA methylome in which each mark is associated with distinct genomic features, potentially allowing the fine-tuning of transcriptional control.

Across the board, 6mA exhibited the highest global methylation entropy, followed by 5hmC and then 5mC. Methylation entropy was also related to genomic context. Specifically, methylation entropy was lowest in the genomic context in which each mark was most common, suggesting that maintenance fidelity is highest in regions where the mark likely has the greatest functional role. In addition, all three marks displayed reduced methylation entropy in sperm cells compared to somatic cells, indicating that methylation levels were more stable in the germline than in the soma. This pattern makes sense because stable DNA methylation is essential for transposable element silencing in the germline, a process that ensures the faithful transmission of genetic information across generations [43,44]. The magnitude of these differences between the soma and the germline varied among marks. In sperm cells, the reduction in methylation entropy was more pronounced for 5mC than for 6mA and 5hmC, suggesting that fidelity maintenance in the germline is more constrained for 5mC than for 6mA and 5hmC. Age-related differences in methylation entropy further supported this pattern. Indeed, even though 6mA and 5mC both displayed significant age-related differences in methylation entropy in somatic cells and sperm cells, it was also the case that absolute methylation entropy was lower in sperm cells than in somatic cells in both age groups. Collectively, the above results revealed that genomic context, sample type, and age all influenced methylation entropy, reflecting differential maintenance fidelity between the soma and the germline and age groups for 6mA, 5mC, and 5hmC. The results also suggest that enzymatic machinery exists for the establishment, maintenance, and erasure of each mark, adding to the evidence that 6mA and 5hmC are bona fide epigenetic marks.

Functional enrichment analyses highlighted the distinct biological functions linked with each mark. 6mA was associated with genes involved in transcription regulation and developmental processes, which has also been seen in insects and mammals [18,42]. 5mC was associated with various processes, proteostasis chief among them. 5hmC was associated with genes regulating neural development and signal transduction, which concurs with findings in mammals [5,6]. The fact that 6mA, 5mC, and 5hmC were linked with distinct biological functions suggests that these methylation types have complementary regulatory roles. Our results, when combined with those from other species, indicate that mark complementarity is conserved across animals. For each methylation mark, a large percentage of the enriched GO terms were shared by somatic cells and sperm cells. Moreover, several GO terms enriched specifically in the soma, the germline, or either age group were involved in similar biological functions. These findings further support the seeming complementarity of 6mA, 5mC, and 5hmC when it comes to maintaining somatic or sperm-specific functions. In sperm specifically, 5mC is crucial for silencing transposable elements [45,46], establishing an epigenomic template for embryo development [47] and genomic imprinting [48]; 5hmC has been associated with the regulation of imprinted genes and sperm maturation processes [49]; 6mA has been linked to genes involved in developmental and metabolic pathways, including fatty acid biosynthesis and PPAR signaling [50]. Because of their complementarity, these methylation marks together form a multilayered epigenetic network essential for sperm function. In addition, age-related differences occurred in each mark’s association with biological functions. In sperm cells, for 6mA and 5hmC, there were fewer age-specific terms and less term overlap, suggesting a reduction in the variety of biological processes that are regulated by these marks with age. In contrast, for 5mC, there were similar term numbers in somatic cells and sperm cells. These findings highlight that the functional consequences of aging on the methylome are dependent on both sample type and mark and that 6mA appeared to be most influenced by age. Recent research conducted under controlled laboratory conditions has shown that 6mA can be used to build epigenetic clocks as accurate as those built using 5mC [22]. Further research is needed under natural conditions to see if 6mA, 5mC, and possibly 5hmC reflect different aging processes.

In conclusion, our findings support a model where the DNA methylome is composed of different, complementary layers of information that are expressed via 6mA, 5mC, and 5hmC, whose involvement in distinct functions may vary across cell types and with age. Each mark was found in different genomic contexts, had different levels of maintenance fidelity, and was associated with different biological functions, suggesting that 6mA, 5mC, and 5hmC play unique yet complementary roles in maintaining cellular homeostasis. While further research will be essential to dissect the mechanistic roles of each mark, our study expands the scope of epigenetics research beyond 5mC, laying the foundation for a more nuanced and integrative understanding of how the DNA methylome encodes epigenetic information in the soma and the germline across time.

## 4. Materials and Methods

### 4.1. Model Organism

The buff-tailed bumblebee (*Bombus terrestris*) was used as a model system because 6mA and 5mC occur at very low levels in insect genomes [51,52], including the *B. terrestris* genome [38]. This species was, thus, ideal for studying different epigenetic marks while simultaneously controlling for mark baseline levels. *B. terrestris* males were obtained from Biobest (Westerlo, Belgium). A total of 150 males from 10 different colonies of origin were randomly assigned to 15 experimental microcolonies, such that each contained 10 males. The microcolonies were maintained under standard laboratory conditions (red light, temperature = 27 ± 1 °C, relative humidity = 50–60%) and were provided with ad libitum access to sugar syrup (Biogluc, Biobest, Westerlo, Belgium).

### 4.2. Sampling

Survival experiments showed that, under laboratory conditions, *B. terrestris* males have an average lifespan of 27.4 ± 7.1 days, with only 3% survival beyond 5 weeks (*n* = 791) [22]. Based on this information, we sampled males from the two extremes of adult lifespan, 1-week-old males (young age group) and 5-week-old males (old age group), to obtain the greatest possible age-related differences. For each age group, somatic cells and sperm cells were sampled. To obtain the somatic cells, males were killed by being flash frozen in liquid nitrogen, and their heads and abdomens were removed to prevent contamination from the pheromonal head glands and gut contents, respectively. To obtain the sperm cells, males were anesthetized with carbon dioxide before being decapitated. For each individual, the abdomen was separated from the thorax and then incisions were made on both sides of the abdomen to expose its contents. The two accessory testes were isolated and washed twice in an insect-specific sperm buffer [53] (semen diluent: 188.3 mM sodium chloride, 5.6 mM glucose, 574.1 nM arginine, 684.0 nM lysine, and 50 mM tris(hydroxymethyl)aminomethane; pH 8.7). Each accessory testis was ruptured in 20 µL of fresh semen diluent to release the sperm into the solution. The overflowing sperm was carefully isolated from the somatic tissues. Samples of somatic and sperm cells were stored at −80 °C until further processing. In total, our dataset consisted of *n* = 13 samples of somatic cells (from 5 young and 8 old individuals) and *n* = 6 samples of sperm cells (from 3 young and 3 old individuals).

### 4.3. DNA Extraction and Library Preparation

High-molecular-weight genomic DNA (gDNA) was extracted from somatic cells or sperm using an in-house SDS/proteinase K protocol. Briefly, the protocol involved lysing the sperm cells using an SDS/proteinase K solution and then performing gDNA purification with phenol–chloroform/chloroform, gDNA precipitation with ethanol and sodium acetate, and assessments of gDNA integrity, quantity, and quality using agarose gel electrophoresis, a Qubit 3.0 fluorometer (Thermo Fisher Scientific, Waltham, MA, USA), and a NanoDrop ONE spectrophotometer (Thermo Fisher Scientific), respectively. gDNA libraries were prepared with the Ligation Sequencing gDNA—Native Barcoding Kit 96 V14 (ONT, SQK-NBD114.96) and sequenced with a PromethION platform (ONT, PRO-SEQ002).

### 4.4. DNA Methylation Analyses

Raw reads were basecalled using Dorado (ONT, v. 0.7.2) and the super-high-accuracy model (‘sup’ command) to capture signals of modified bases (6mA, 5mC, and 5hmC) across all genomic contexts. Basecalled reads were aligned to the *B. terrestris* reference genome (GCF_910591885.1 v. 1.2) using Dorado (‘aligner’ command). Genome-wide methylation levels were determined for each mark using Modkit (‘summary’ command; ONT, v. 0.3.2). Genome-wide, base-resolution levels for 6mA, 5mC, and 5hmC were determined using Modkit (‘pileup’ command; ONT, v. 0.3.2). Only sites with a minimum coverage of 10X were retained in the subsequent analyses. Bases were considered to be effectively methylated when the methylated fraction exceeded 0.7 [54]. BEDTools [55] was used to determine the genomic context of each methylated site, and then genomic distributions were ascertained for each mark. Methylation entropy was determined using the average site-specific methylation entropy per sample and per mark [33]. For the functional enrichment analyses, we selected gene ontology (GO) terms that effectively linked methylated sites with biological processes (BPs), molecular functions (MFs), and cellular components (CCs). The GO term data were downloaded from the Hymenoptera Genome Database [56]. The functional enrichment analyses were conducted using the topGO package [57] (v. 2.50.0).

### 4.5. Statistical Analyses

All statistical analyses were conducted in RStudio (Version 2023.06.0+421). Genome-wide methylation levels were compared using non-parametric Kruskal–Wallis tests when comparing >2 groups, followed by Dunn’s post hoc tests with Benjamini–Hochberg correction for multiple comparisons. For pairwise comparisons between two groups, Wilcoxon Mann–Whitney tests were used unless assumptions of normality and homoscedasticity were met, in which case Student’s *t*-tests (equal variance) or Welch’s *t*-tests (unequal variance) were applied. Normality and variance homogeneity were assessed using the Shapiro–Wilk and Levene’s tests, respectively.

Differences in genomic distributions of methylated sites across contexts (exons, introns, promoters, intergenic regions, gene bodies) were tested using permutational multivariate analysis of variance (PERMANOVA) with 999 permutations. Significant effects were further explored with pairwise post hoc PERMANOVA tests with FDR correction, as implemented in the vegan R package v 2.7-1 [58].

Methylation entropy values were analyzed using the same framework (Kruskal–Wallis, ANOVA, *t*-test/Wilcoxon, depending on design and assumptions) to test for differences among marks, between sample types, and across age groups.

GO enrichment analyses were conducted separately for BP, MF and CC. The ‘weight01’ algorithm was applied with Fisher’s exact test; GO terms were considered significantly enriched at FDR < 0.05 (Benjamini–Hochberg correction).

All *p*-values reported in the Results are adjusted for multiple corrections where applicable.

## Data Availability

The original data presented in the study are openly available in the SRA repository. GO terms enrichment analysis results can be found on Figshare (doi: 10.6084/m9.figshare.30085363).

## Supplementary Materials

The following supporting information can be downloaded at: https://www.mdpi.com/article/10.3390/epigenomes9030035/s1, Figure S1. Entropy levels comparison between methylation types across genomic contexts. The line through the middle of each box is the median, and the lower and upper edges are the first and third quartiles, respectively. The different letters above the boxes indicate statistically significant differences (Kruskal–Wallis test: *p* < 0.05 and Dunn’s post-hoc test: *p* < 0.05); Figure S2. Entropy levels comparisons between somatic tissues and sperm cells across genomic contexts. The line through the middle of each box is the median, and the lower and upper edges are the first and third quartiles, respectively. Statistical significance was tested with pairwise Wilcoxon Mann–Whitney tests (* *p* < 0.05, ** *p* < 0.01, *** *p* < 0.005); Figure S3. Entropy levels comparisons between somatic tissues and sperm cells, and ages across genomic contexts. The line through the middle of each box is the median, and the lower and upper edges are the first and third quartiles, respectively. Statistical significance was tested with Student’s *t*-test, Welch’s test in case of unequal variance, or pairwise Wilcoxon Mann-Whitney in case of non-normal distribution (* *p* < 0.05, ** *p* < 0.01, *** *p* < 0.005).

## Abbreviations

The following abbreviations are used in this manuscript:

5mC	C5-methylcytosine
6mA	N6-methyadenine
5hmC	C5-hydroxymethylcytosine
GO	Gene ontology
