# Supplementary material for: Comparative Mapping of N6-Methyladenine, C5-Methylcytosine, and C5-Hydroxymethylcytosine in a Single Species Reveals Constitutive, Somatic- and Germline-Specific, and Age-Related Genomic Context Distributions and Biological Functions
Source: Epigenomes. 2025 Sep 18;9(3):35. doi: 10.3390/epigenomes9030035 (PMC12452632; doi:10.3390/epigenomes9030035)
Supplement: Supplementary file 1 [file epigenomes-09-00035-s001.zip › epigenomes-3864811-supplementary.pdf]

epigenomes

Supplementary Figures

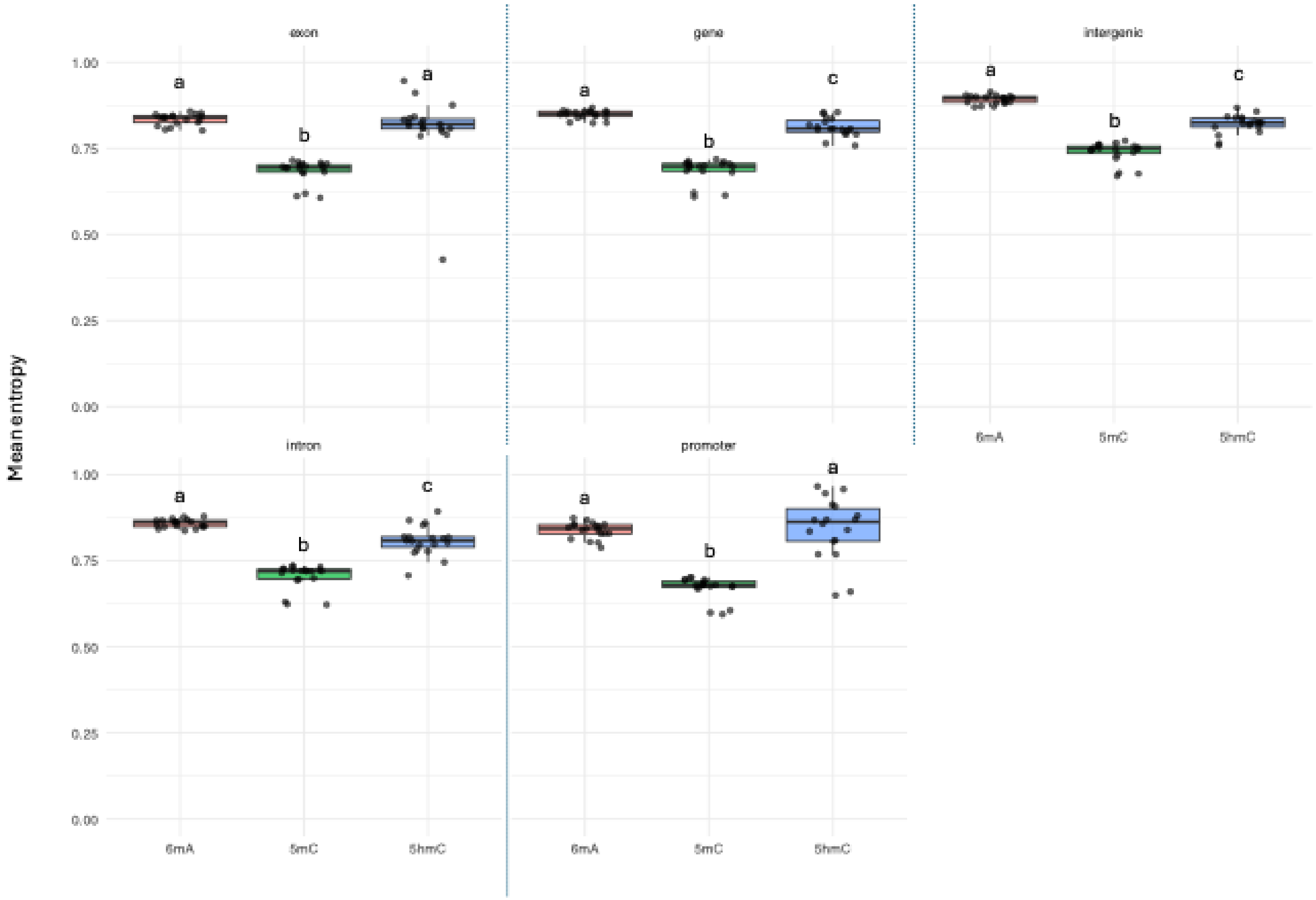

Figure S1. Entropy levels comparison between methylation types across genomic contexts. The line through the middle of each box is the median, and the lower and upper edges are the first and third quartiles, respectively. The different letters above the boxes indicate statistically significant differences (Kruskal–Wallis test: $p < 0.05$ and Dunn's post-hoc test: $p < 0.05$).

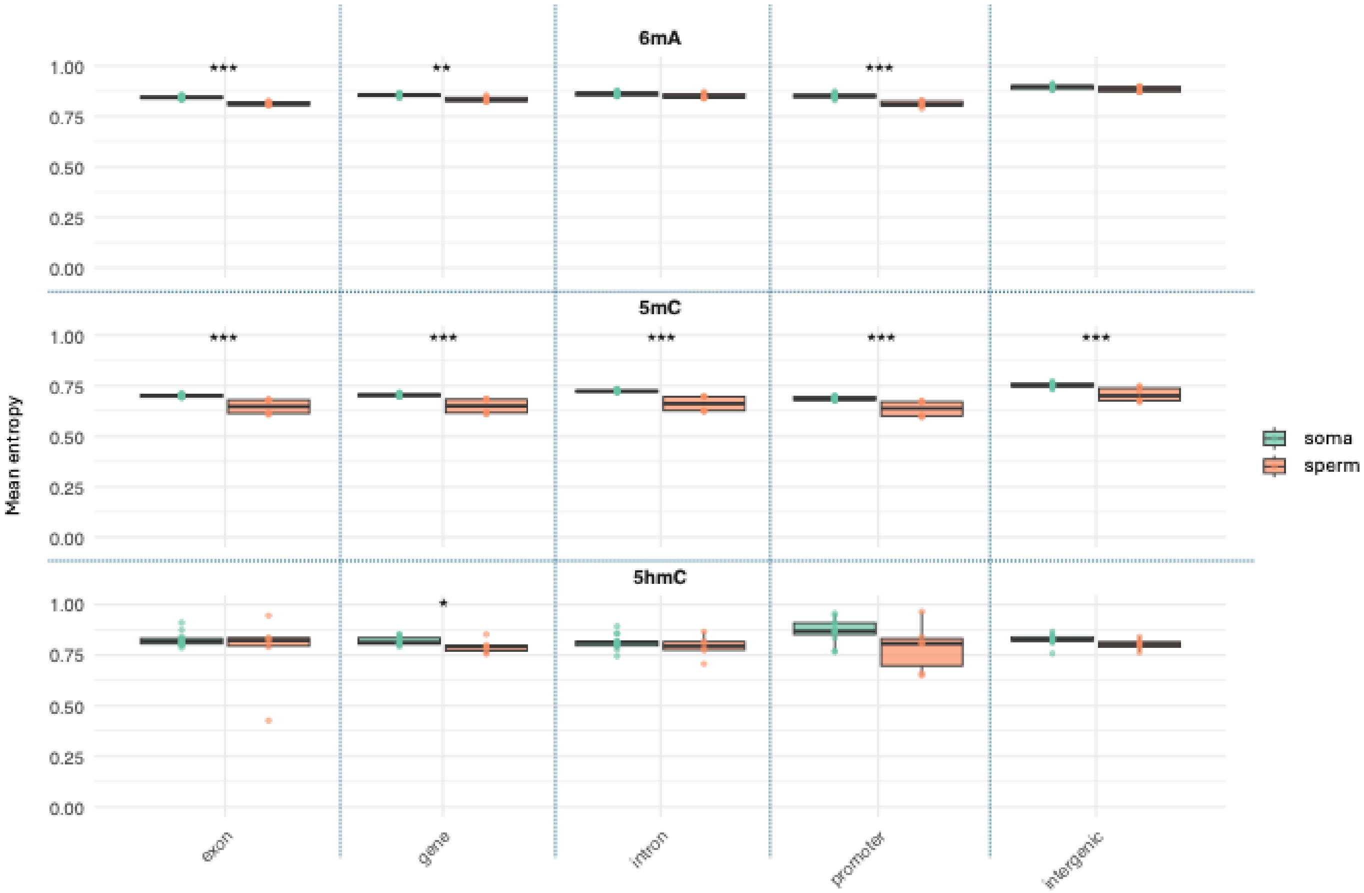

Figure S2. Entropy levels comparisons between somatic tissues and sperm cells across genomic contexts. The line through the middle of each box is the median, and the lower and upper edges are the first and third quartiles, respectively. Statistical significance was tested with pairwise Wilcoxon Mann–Whitney tests (*$p < 0.05$, ** $p < 0.01$, *** $p < 0.005$).

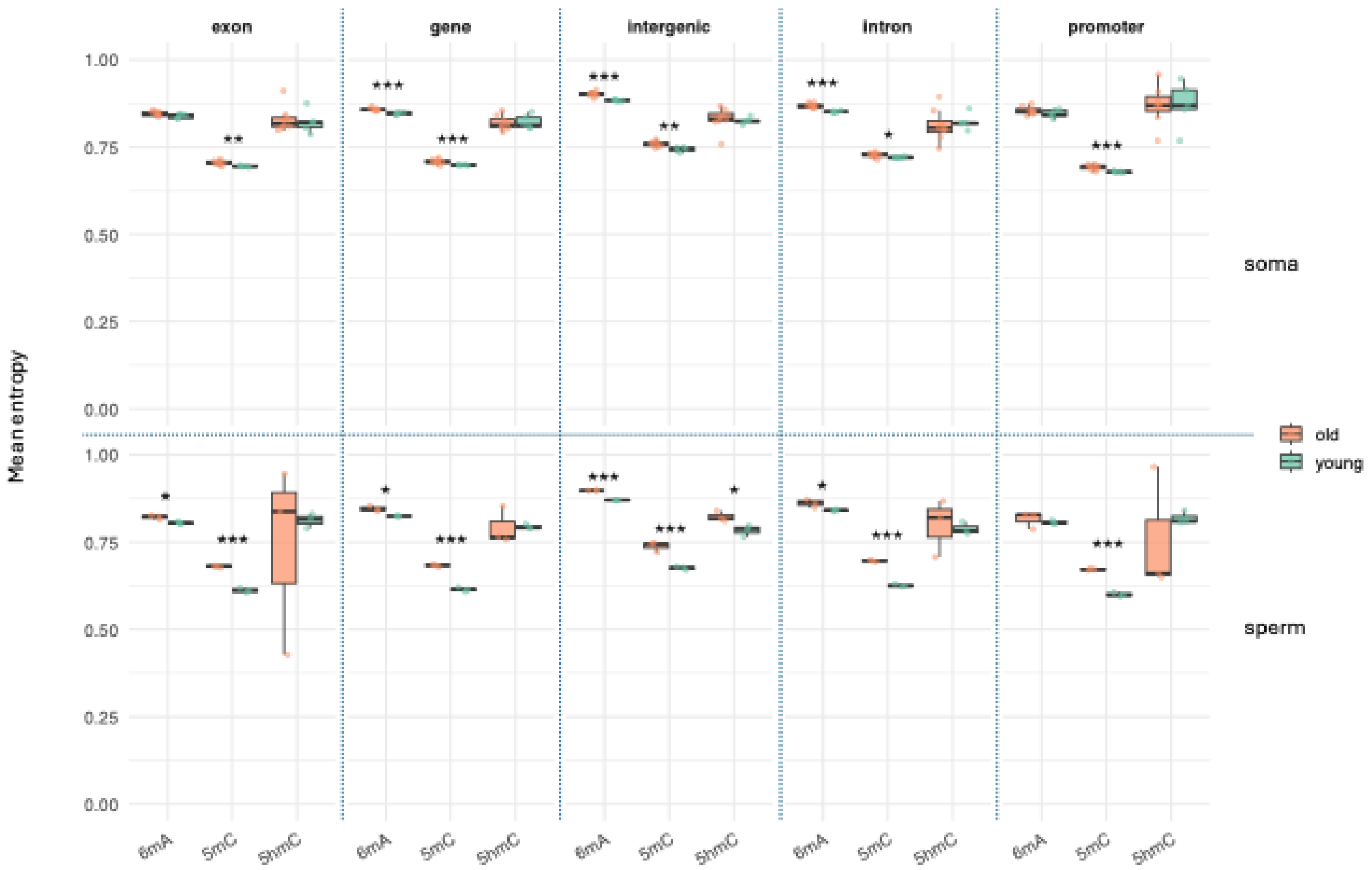

Figure S3. Entropy levels comparisons between somatic tissues and sperm cells, and ages across genomic contexts. The line through the middle of each box is the median, and the lower and upper edges are the first and third quartiles, respectively. Statistical significance was tested with Student's *t*-test, Welch's test in case of unequal variance, or pairwise Wilcoxon Mann-Whitney in case of non-normal distribution (*$p < 0.05$, ** $p < 0.01$, *** $p < 0.005$).
